# Patient and Public Involvement in Inflammatory Bowel Disease Research—A Scoping Review

**DOI:** 10.1093/jcag/gwad054

**Published:** 2023-12-14

**Authors:** Karam Elsolh, Amy Li, Malini Hu, Samir Seleq, Emma Neary, Nikko Gimpaya, Michael A Scaffidi, Teruko Kishibe, Rishad Khan, Samir C Grover

**Affiliations:** Michael G. DeGroote School of Medicine, Faculty of Health Sciences, McMaster University, 1280 Main St W, Hamilton, Ontario, Canada, L8S 4L8; Division of Gastroenterology, St. Michael’s Hospital, University of Toronto, 36 Queen St E, Toronto, Ontario, Canada, M5B 1W8; Division of Gastroenterology, St. Michael’s Hospital, University of Toronto, 36 Queen St E, Toronto, Ontario, Canada, M5B 1W8; Division of Gastroenterology, St. Michael’s Hospital, University of Toronto, 36 Queen St E, Toronto, Ontario, Canada, M5B 1W8; Department of Medicine, University of Toronto, 6 Queen’s Park Crescent W, Toronto, Ontario, Canada, M5S 3H2; Division of Gastroenterology, St. Michael’s Hospital, University of Toronto, 36 Queen St E, Toronto, Ontario, Canada, M5B 1W8; Division of Gastroenterology, St. Michael’s Hospital, University of Toronto, 36 Queen St E, Toronto, Ontario, Canada, M5B 1W8; Division of Gastroenterology, St. Michael’s Hospital, University of Toronto, 36 Queen St E, Toronto, Ontario, Canada, M5B 1W8; Li Ka Shing Knowledge Institute, Unity Health Toronto, 209 Victoria St, Toronto, Ontario, Canada, M5B 1T8; Division of Gastroenterology, St. Michael’s Hospital, University of Toronto, 36 Queen St E, Toronto, Ontario, Canada, M5B 1W8; Department of Medicine, Queen’s University, 94 Stuart Street, Kingston, Ontario, Canada, K7L 3N6; Li Ka Shing Knowledge Institute, Unity Health Toronto, 209 Victoria St, Toronto, Ontario, Canada, M5B 1T8; Division of Gastroenterology, St. Michael’s Hospital, University of Toronto, 36 Queen St E, Toronto, Ontario, Canada, M5B 1W8; Li Ka Shing Knowledge Institute, Unity Health Toronto, 209 Victoria St, Toronto, Ontario, Canada, M5B 1T8

## Abstract

**Background:**

Interest in patient and public involvement in research has grown. Medical, health, and social care research has demonstrated several benefits of patient and public engagement, such as empowering user input and reducing attrition rates in clinical trials. To date, no study has reviewed patient engagement in inflammatory bowel disease (IBD). We aimed to describe the benefits, challenges, and best practices of patient engagement in IBD research.

**Methods:**

We performed a systematic search on MEDLINE, EMBASE, and Cochrane for all clinical IBD research studies in which patients were involved in the research process (1946- 2023). Patient input was considered in: (1) study design, (2) study execution, (3) research dissemination, and/or (4) other domains not specified here. Two authors independently screened and extracted data on type of engaged person(s), format of engagement, author-reported benefits, recommendations, and challenges. For each study, we reported the level of patient engagement and study adherence to standardized reporting guidelines.

**Results:**

After screening 9,355 articles, we included 51 for final analysis. IBD patients were most frequently engaged in study design. Patient engagement in IBD research improved recruitment rates and promoted the creation of user-friendly quality-of-life tools. Selection bias and recruitment difficulties were common challenges in the application of patient engagement. Authors recommended continuous patient involvement to address emerging priorities and cognitive interviewing to improve questionnaire clarity.

**Conclusions:**

Patient engagement represents an important step in promoting patient-centred care. According to study authors, implementing cognitive interviewing techniques, continuous patient involvement, and standardized reporting guidelines may improve future iterations of engagement in IBD.

## Introduction

Interest in patient and public involvement (PPIn) in research has grown.^1^ An early example of patient involvement occurred in the 1980s in the UK, wherein patient group representatives conducted a trial on chorionic villous sampling in pregnancy.^2^ Stakeholder engagement has also been adopted in social and healthcare research in the UK, where the INVOLVE project supports active public involvement in the National Health Service (NHS).^3^ Since then, there has been a growing emphasis on engaging patients in research directly related to their care.^4^

Research in health and social care has demonstrated several benefits of PPIn.^5,6^ These include empowering user input and providing researchers with a greater understanding of community perspectives.^6^ In medicine, PPIn has been demonstrated to positively impact patient enrolment in clinical trials.^1^ On ethical grounds, engaging patients in research relevant to their care may democratize the research process.^7^ To date, no study has comprehensively reviewed patient engagement in inflammatory bowel disease (IBD) research. IBD is a chronic disease with a significant burden on patients’ personal, social, and occupational functioning.^8^ IBD patients frequently play an active role in their disease management.^8^ As such, IBD patients may be able to offer their knowledge to research studies as experience-based experts.

In this scoping review, we analyzed studies that actively involved IBD patients (and other relevant stakeholders) in research. Using a meta-narrative approach,^9^ we aimed to provide a synthesis on implementing patient engagement in IBD and to address the following questions: How and during what stage of research are IBD patients engaged? What are author-reported recommendations for best practices of PPIn in IBD? What are the benefits of PPIn in IBD? What are the challenges (and suggested solutions) of PPIn in IBD?

## Methods

We comprehensively reviewed IBD research studies in which patients were active participants in the research process. Reporting of our findings followed guidelines for the reporting of scoping reviews (PRISMA extension for Scoping Reviews).^10^ A completed checklist of the reporting guidelines can be found in Text, Supplementary Data Content 1.

### Search strategy

We ran a systematic search in EMBASE, Medline, and the Cochrane Central Register of Controlled Trials for clinical IBD research studies in which patients were actively involved in research. The following study types were considered for inclusion: clinical practice guidelines, randomized controlled trials, cohort studies, cross-sectional studies, systematic reviews, meta-analyses, survey studies, cost-effectiveness analyses, and qualitative studies. We excluded opinion pieces, editorials, abstract-only submissions, research letters, case reports/series, and protocol-only entries. We only included articles published in English. The search was not limited to specific countries. The search was designed by a librarian. The search strategy was also peer-reviewed and assessed against the Peer Review of Electronic Search Strategy (PRESS) checklist.^11^ The searches were run from inception to August 21, 2023. The full search strategies can be found in Text, Supplementary Data Content 2.

### Study selection

Two authors independently reviewed the abstracts (K.E., E.N.) and full texts (K.E., A.L.) for eligible studies using the screening platform Covidence. Discrepancies were resolved by consensus. Patient input was considered in: study design (prioritization of research topics, outcome selection, study tool development, and funding acquisition); study execution (study recruitment, data collection, and analysis); dissemination of research (manuscript development); and/or other domains not specified here. Engaged person(s) included IBD patient(s), and/or other stakeholders (patient representatives, caregivers, and community members).

### Data extraction

Two authors extracted the following data from full texts: article type, study objectives, research setting, enrolment method(s), sampling method(s), engaged person(s), and method(s) of PPIn, which included focus groups, semi-structured interviews, surveys, and/or multidisciplinary panels. Where possible, we sorted instances of PPIn into one or more of the following categories: study design, study execution, and/or dissemination of results.

For each instance of PPIn, we obtained a narrative description of patient contributions from the study article. We searched each full text for author and patient-reported best practices, benefits, challenges (and suggested solutions) of PPIn. We reported level of patient engagement according to the Patient-Oriented Research Level of Engagement Tool (PORLET) and adherence to standardized reporting guidelines according to the Guidance for Reporting Involvement of Patients and the Public, Version 2 (GRIPP2) for each study.^12,13^

### Analysis

Quantitative data on article type, engaged person(s), method(s) of engagement, and stage of patient engagement in the research process was summarized using descriptive statistics. The qualitative data were analyzed using a meta-narrative approach, adopted from Domecq et al.^7,9^ Following a literature search with clear selection criteria, the approach adheres to a framework defining four key questions (methods, best practices, benefits, and challenges of PPIn). As per the framework, we reviewed narrative data from each article and synthesized key themes, using specific studies to highlight themes and map available evidence. Specifically, we reviewed the Discussion section of each article for methods, benefits, challenges, and best practices of PPIn as identified from prior literature,^7,14^ and extracted any additional themes reported by the authors.

## Results

**Figure 1.**
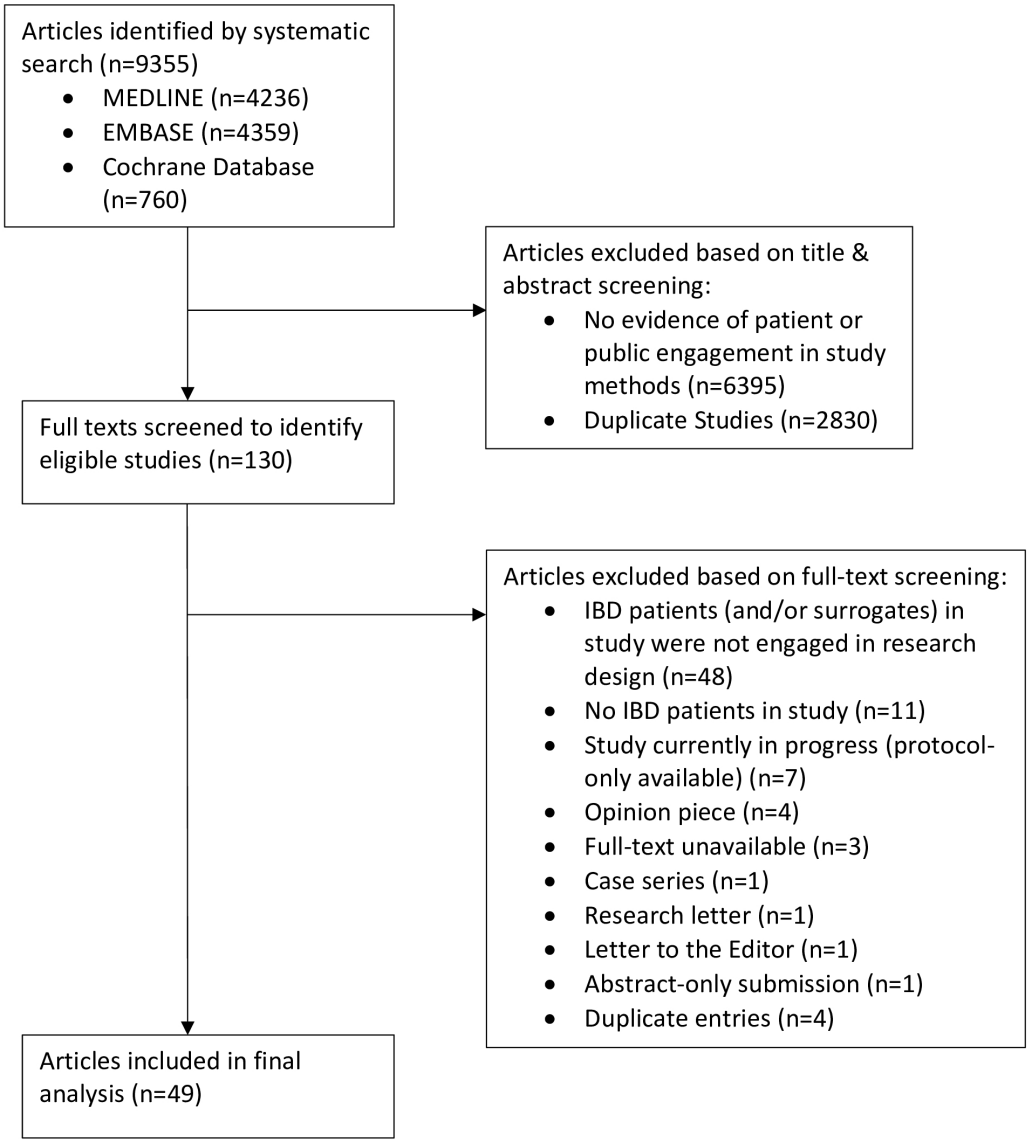
Study flow chart.

Our search identified 9,355 articles. Following title and abstract screening and full-text review, we included 51 articles (Figure 1). Most articles were qualitative studies (*n* = 41, 80.4%). Our sample also included four survey studies (7.8%), two randomized controlled trials (RCTs) (3.9%), three retrospective cohort studies (5.9%), and one cross-sectional study (2.0%). Thirty-three percent (*n* = 16) of patient engagement studies were based in the UK, and the majority (*n* = 48, 94.1%) were academic-affiliated.

Most studies involved IBD patients directly (*n* = 50, 98%). Remaining engaged parties included patient representatives (*n* = 14, 27.4%), caregivers (*n* = 10, 19.6%), and community members (*n* = 1, 2.0%). Patients were engaged via semi-structured interviews (*n* = 24, 47.1%), multi-disciplinary panels (*n* = 27, 52.9%), focus groups (*n* = 22, 43.1%), and surveys (*n* = 13, 25.5%). Patients were engaged in study design in most studies (*n* = 50, 98%), study execution in 22 studies (43.1%), and dissemination of results in nine studies (17.6%). A summary of article characteristics can be found in Table 1.^15–65^

**Table 1.** Study characteristics and level of engagement according to Patient-Oriented Research Level of Engagement Tool (PORLET) criteria (*n* = 51).

Article	Article Year	Article objective	Article type	Study institution (country)	Study institution (type)	Engaged person(s)	Method of engagement	Timing of engagement (study Phase)	PORLET Score (/25)
Adegbola et al.^15^	2021	Patient-reported outcome measures	Qualitative study	United Kingdom	Academic hospital	Patient, patient representative	Semi-structured interviews	Study design, study execution	14
Adegbola et al.^16^	2020	Thematic analysis	Qualitative study	United Kingdom	Academic hospital	Patient, patient representative	Multi-disciplinary panels	Study design	12
Adler et al.^17^	2016	Thematic analysis	Qualitative study	United States	Academic hospital	Patient, caregiver	Surveys, tool development	Study design	11
Alnaqbi et al.^18^	2013	IBD instrument development	Qualitative study	Canada	Academic hospital	Patient	Surveys, semi-structured interviews	Study design	7
Alrubaiy et al.^19^	2015	IBD instrument development	Qualitative Study	United Kingdom	Academic hospital	Patient	Surveys, semi-structured interviews, focus groups	Study design	8
Bitton et al.^20^	2020	IBD instrument development	Qualitative Study	Canada	Academic hospital	Patient, patient representatives	Focus groups, multi-disciplinary panels	Study design	8
Bitton et al.^21^	2019	Quality indicator assessment	Qualitative study	Canada	Academic hospital	Patient, patient representative	Focus groups, multi-disciplinary panels	Study design	10
Bodger et al.^22^	2014	IBD instrument development, IBD instrument validation	Qualitative study	United Kingdom	Academic hospital	Patient	Focus groups, semi-structured interviews	Study design	11
Carter et al.^23^	2020	Thematic analysis	Qualitative study	United Kingdom	Academic hospital	Patient, caregiver	Surveys, semi-structured interviews	Study design, study execution, dissemination of results	14
Casellas et al.^24^	2013	IBD instrument Development, IBD instrument Validation	Qualitative study	Spain	Academic hospital	Patient	Semi-structured interviews	Study design	7
Cheifetz et al.^25^	2012	IBD research priorities	Qualitative study	United States	Academic hospital	Patient	Focus groups	Study design	10
de Jong et al.^26^	2017	IBD instrument development	Qualitative study	Netherlands	Academic hospital	Patient	Multi-disciplinary panels	Study design	17
Denters et al.^27^	2012	IBD instrument development	Qualitative study	Netherlands	Academic hospital	Patient	Focus groups	Study design	9
Dibley et al.^28^	2018	IBD instrument development, IBD instrument validation	Qualitative study	United Kingdom	Academic hospital	Patient	Semi-structured interviews	Study design, study execution	14
Dibley et al.^29^	2020	IBD instrument development, qualitative analysis	Qualitative study	United Kingdom	Academic hospital	Patient	Multi-disciplinary panels	Study design, study execution, dissemination of results	14
Fofaria et al.^30^	2019	Quality improvement project	Qualitative study	United Kingdom	Academic hospital	Patient, patient representative	Multi-disciplinary panels	Study design, study execution	20
Grant et al.^31^	2019	IBD research priorities	Qualitative study	Canada	Academic hospital	Patient, caregiver	Focus groups, multi-disciplinary panels	Study design, study execution	23
Guida et al.^32^	2021	Thematic analysis	Qualitative study	Italy	Academic hospital	Patient	Focus groups	Study design	7
Haaland et al.^33^	2014	IBD instrument development, IBD instrument validation	Qualitative study	Canada	Academic hospital	Patient, caregiver	Focus groups, semi-structured interviews	Study design	7
Hart et al.^34^	2017	IBD research priorities	Qualitative study	United Kingdom	Academic hospital	Patient, patient representative	Multi-disciplinary panels	Study design, study execution	17
Hubbard et al.^35^	2019	IBD instrument development	Qualitative study	United Kingdom	Academic hospital	Patient	Multi-disciplinary panels	Study design, study execution, dissemination of results	16
Hughes et al.^36^	2016	IBD instrument development, IBD instrument validation	Survey with high response rate	United Kingdom	Academic hospital	Patient	Semi-structured interviews	Study design	13
Kapasi et al.^37^	2020	IBD instrument development	Qualitative study	United Kingdom	Academic hospital	Patient, patient representative, caregiver	Focus groups, multi-disciplinary panels	Study design	16
Katarina et al.^38^	2018	IBD instrument development	Qualitative study	Sweden	Academic hospital	Patient	Surveys, semi-structured interviews, focus groups	Study design	10
Kennedy et al.^39^	2003	IBD instrument development	Intervention studies—RCT	United Kingdom	Academic hospital	Patient, patient representative	Focus groups, multi-disciplinary panels	Study design	13
Kennedy et al.^40^	2002	IBD instrument development	Qualitative study	United Kingdom	Academic hospital	Patient	Semi-structured interviews	Study design	11
Kennedy et al.^41^	2019	IBD instrument development	Qualitative study	United States	Academic hospital	Patient, patient representative	Multi-disciplinary panels	Study design, study Execution, dissemination of results	22
Khalil et al.^42^	2020	IBD instrument development	Qualitative study	United States	Academic hospital	Patient	Semi-structured interviews, focus groups	Study design	13
Kim et al.^43^	2021	IBD unstrument Development	Qualitative study	Australia	Academic hospital	Patient	Surveys, semi-structured interviews, focus groups, multi-disciplinary panels	Study design	10
Kim et al.^44^	2018	IBD Instrument Development	Qualitative study	Australia	Academic hospital	Patient	Semi-structured interviews, focus groups, multi-disciplinary panels	Study design	20
Lee et al.^45^	2021	IBD Instrument Development	Qualitative study	United Kingdom	Academic hospital	Patient, patient representative	Surveys, semi-structured interviews, multi-disciplinary panels	Study design	8
Louis et al.^46^	2020	IBD Research Priorities	Qualitative study	Belgium	Academic hospital	Patient, patient representative	Focus groups, multi-disciplinary panels	Study design, study execution	17
Wickman et al.^47^	2018	IBD Instrument Development	Qualitative study	Sweden	Academic hospital	Patient	Surveys, semi-structured interviews	Study design, study execution	8
Macdonald et al.^48^	2018	Thematic Analysis	Observational studies—Cross-sectional study	Canada	Academic hospital	Patient	Surveys, multi-disciplinary panels	Study design, study execution	17
Marín-Jiménez et al.^49^	2017	IBD Instrument Development	Survey with high response rate	Spain	Academic hospital	Patient, patient representative	Surveys, focus groups, multi-disciplinary panels	Study design, study execution	17
McDermott et al.^50^	2018	Patient Education in IBD	Qualitative study	Ireland	Academic hospital	Patient	Surveys, focus groups	Study design, Study execution	12
Oliver et al.^51^	2021	IBD Instrument Development	Qualitative study	United States	Academic hospital	Patient	Multi-disciplinary panels	Study design	9
Rohatinsky et al.^52^	2020	Thematic Analysis	Qualitative study	Canada	Community hospital	Patient, caregiver	Semi-structured interviews, multi-disciplinary panels	Study design, study execution, dissemination of results	25
Ruan et al.^53^	2017	IBD instrument development, IBD instrument validation	Qualitative study	China	Academic hospital	Patient	Surveys, semi-structured interviews	Study design	8
Vent-Schmidt et al.^54^	2020	Thematic analysis	Qualitative study	Canada	Academic hospital	Patient	Semi-structured interviews, focus groups	Study design	8
Vergara et al.^55^	2002	IBD instrument development, IBD instrument validation	Survey with high response rate	Spain	Academic hospital	Caregiver, community member	Semi-structured interviews	Study design	7
Williams et al.^56^	2021	IBD instrument development	Qualitative study	Australia	Academic hospital	Patient, patient representative	Surveys, semi-structured interviews, focus groups	Study design	18
Sahnan et al.^57^	2019	Patient-reported outcome measures	Qualitative study	United Kingdom	Academic hospital	Patient, patient representative	Semi-structured interviews, multi-disciplinary panels	Study design, study execution	13
Heisler et al.^58^	2023	Thematic analysis	Qualitative study	Canada	Community hospital	Patient	Multi-disciplinary panels	Study design, study execution	16
Santos et al.^59^	2022	Health systems research	Observational studies—cohort study	Canada	Community hospital	Patient, caregiver	Multi-disciplinary panels	Study design, study execution, Dissemination of results	20
Cavallaro et al.^60^	2021	Patient-reported outcome measures	Qualitative study	New Zealand	Academic hospital	Patient	Focus groups, multi-disciplinary panels	Study execution	7
Rines et al.^61^	2021	Thematic analysis	Qualitative study	Canada	Academic hospital	Patient	Multi-disciplinary panels	Study design, study execution, dissemination of results	25
Long et al.^62^	2023	IBD instrument development	Intervention studies—RCT	United States	Academic hospital	Patient	Semi-structured interviews, focus groups	Study design	8
Gorbenko et al.^63^	2021	IBD instrument development	Qualitative study	United States	Academic hospital	Patient	Focus groups	Study design	8
Peña-Sánchez et al.^64^	2022	Prevalence study	Observational studies—Cohort study	Canada	Academic hospital	Patient, caregiver	Multi-disciplinary panels	Study design, dissemination of results	20
Peña-Sánchez et al.^65^	2022	Health systems research	Observational studies—Cohort study	Canada	Academic hospital	Patient, Caregiver	Semi-structured interviews, multi-disciplinary panels	Study design, study execution, dissemination of results	25

### How are IBD patients engaged in the research process?

The most frequent form of PPIn was patient consultation in the development of IBD study tools, including questionnaires, guidebooks, and a video animation (*n* = 44, 86.3%). A qualitative study exploring disclosure of IBD among children utilized a core e-Advisory group of IBD patients to provide insight into young people’s experiences with disclosure,^23^ which informed the development of a two-minute animation.^23^

IBD patients and their surrogates also acted as active participants in study recruitment in four studies (7.8%).^15,35,52,65^ In one study, patient champions within the project team devised a new method of identifying and transferring IBD patients to specialist nurse-led telephone clinics, which helped boost transfer rates.^30^

Other modalities of PPIn included assisting with data analysis (*n* = 8, 15.7%), wherein one study provided patients with training in qualitative analysis methods from an experienced qualitative researcher.^29^ Six studies (11.8%) included patients and/or other stakeholders (e.g., patient advisors) as co-authors on the published work. Of note, one study on the development of an IBD transfer toolkit was spearheaded by an IBD patient, who acted as the lead study author in collaboration with a paediatric gastroenterologist.^41^ This was achieved by involving the patient partner from the outset of the project in study design. One retrospective study directly cited engaged patients’ reflections on study results in their discussion, including their thoughts and assessment of the increasing prevalence of IBD among First Nations.^64^

Adherence to reporting guidelines for PPIn was evaluated via the GRIPP2 checklist (**Table 2**). All studies in our cohort (*n* = 51) reported the methods used for PPIn. Twenty-five reported the aim of PPIn, 35 reported results of PPIn, 32 reported positive and/or negative effects of PPIn, and 19 commented critically on PPIn in the study (reflecting on positive experiences and/or areas of improvement).

**Table 2.** PPIn according to guidance for reporting involvement of patients and the public, version 2 (GRIPP2) reporting checklist (short form) (*n* = 49).

Article	1. Aim	2. Methods	3. Results	4. Discussion/conclusions	5. Reflections/critical perspective
Adegbola et al.^15^		X	X	X	
Adegbola et al.^16^	X	X	X	X	
Adler et al.^17^		X			
Alnaqbi et al.^18^		X	X		
Alrubaiy et al^19^		X	X		
Bitton et al.^20^	X	X	X		
Bitton et al.^21^		X	X	X	X
Bodger et al.^22^	X	X	X	X	X
Carter et al.^23^		X	X		
Casellas et al.^24^		X	X	X	X
Cheifetz et al.^25^		X			X
de Jong et al.^26^		X			
Denters et al.^27^	X	X	X	X	X
Dibley et al.^28^	X	X	X	X	
Dibley et al.^29^		X	X	X	
Fofaria et al.^30^		X			X
Grant et al.^31^	X	X	X	X	X
Guida et al.^32^		X			
Haaland et al.^33^		X			
Hart et al.^34^	X	X	X	X	X
Hubbard et al.^35^	X	X			
Hughes et al.^36^		X	X	X	
Kapasi et al.^37^	X	X	X	X	
Katarina et al.^38^	X	X	X	X	X
Kennedy et al.^39^		X	X	X	
Kennedy et al^40^	X	X		X	
Kennedy et al.^41^	X	X	X	X	
Khalil et al.^42^	X	X	X	X	X
Kim et al.^43^		X	X	X	
Kim et al.^44^	X	X	X	X	X
Lee et al.^45^		X			
Louis et al.^46^		X	X	X	X
Wickman et al.^47^		X	X	X	X
Macdonald et al.^48^	X	X		X	X
Marín-Jiménez et al.^49^		X	X	X	
McDermott et al.^50^	X	X	X	X	
Oliver et al.^51^		X	X	X	
Rohatinsky et al.^52^	X	X	X	X	
Ruan et al.^53^		X			
Vent-Schmidt et al.^54^	X	X			
Vergara et al.^55^		X	X	X	
Williams et al.^56^	X	X			X
Sahnan et al.^57^		X	X	X	X
Heisler et al.^58^	X	X			
Santos et al.^59^		X	X	X	
Cavallaro et al.^60^	X	X	X	X	
Rines et al.^61^	X	X	X	X	X
Long et al.^62^	X	X			
Gorbenko et al.^63^		X	X	X	X
Peña-Sánchez et al.^64^	X	X	X		X
Peña-Sánchez et al.^65^	X	X			

### Level of engagement

Level of patient engagement was reported for each study according to the PORLET criteria, which gauges the degree of PPIn according to five criteria (Table 1). Score breakdown by individual criteria is available in Text, Supplementary Data Content 3. The mean total PORLET score was 13.3 (SD 5.4). There was a wide range of PORLET scores reported,^7–25^ pointing to heterogeneity in the level of engagement.

A pair of studies linked to a patient-oriented research initiative achieved a high level of engagement.^52,65^ One study evaluated perspectives of healthcare use among rural Canadians with IBD.^52^ The study engaged patient and family advisors as equal members in decision-making. This was achieved by engaging patients in all phases of the research process. Patient partners contributed to the study design, co-created recruitment materials, reviewed findings, and co-developed the manuscript.^52,65^ A separate study with high patient engagement compared healthcare utilization between First Nations patients and the general IBD population. This retrospective cohort study involved indigenous patients in study outcome selection, data analysis, and the knowledge-sharing phases of research (wherein 1 advocate co-presented the published work at multiple conferences).^59^ This was mediated by sending the engaged parties periodic reports and requesting regular feedback on results.^59^

### What are author-reported recommendations for best practices of PPIn in IBD?

Three IBD studies in our sample (5.9%) cited the importance of recruiting patients broadly for PPIn.^15,24,36^ In one study, authors noted that recruiting patients from a wide variety of centres and different regions of Spain helped ensure the generalizability of their findings.^29^ Two studies (3.9%) encouraged cognitive interviewing with patients as a tool in questionnaire development, with a validation study noting it improved question clarity, ease of understanding, and increased item validity.^38,47^ Last, a study engaging IBD patients in online survey development recommended continuous patient involvement to address patients’ emerging concerns during clinical testing.^54^

### What are the benefits of PPIn in IBD?

The most reported benefit of PPIn, cited across 21 studies (41.2%), was that it helped promote patient-centred care and/or ensured that the patient voice was represented. In one study soliciting research uncertainties in the management of pediatric IBD, authors highlighted the role of patient engagement in establishing a research agenda that was mutually impactful to both those treating and those experiencing the disease.^31^

A second theme centred on the role of PPIn in strengthening the relevance and/or credibility of study items. Authors of a quality-of-life questionnaire for household members of IBD stated that involving IBD stakeholders in the development of their tool helped focus items on issues most relevant to responders.^55^ Lastly, a tertiary benefit reported by one study was that patient engagement promoted the creation of a user-friendly and understandable quality-of-life tool in IBD research.^19^

### What are the challenges (and suggested solutions) of PPIn in IBD?

Two studies (3.9%) cited feasibility as a challenge to incorporating PPIn in IBD research, specifically as it relates to recruiting and engaging participants as part of study design.^25,56^ In a study testing a decision aid encompassing reproductive decisions in IBD, authors made note of intrinsic difficulties with patient recruitment for PPIn, related to the time required to conduct patient focus groups and guide the development of the decision aid. This was cited as a challenge in recruiting patient participants, especially young patients who may have professional commitments and/or time constraints related to family.^56^ Another challenge identified across 4 studies (7.8%) was that engaged patients may not be representative of the entire IBD population. One study highlighted a lack of diversity in education and literacy among focus group participants.^42^ The authors of a quality-of-care questionnaire developed by Swedish IBD patients stated that the use of the questionnaire may be limited to a Swedish care context.^38^ These authors recommended cultural adaptation of the tool if it were to be used in a different context.

## Discussion

In our analysis of 51 original articles involving patients and/or other stakeholders in IBD research, we found that IBD patients were most frequently engaged in the design phase of the research process. Across three studies, they were involved in setting IBD research priorities. The influence of PPIn on the research agenda has previously been reported in a review of INVOLVE, a publicly funded NHS patient involvement project.^14^ Several studies in the review demonstrated that PPIn led to clearer research priorities, a wider set of topics being considered, and a shift in research focus to be more in line with public concerns.^14,66^ More recently, a summative report on the impact of IBD in Canada incorporated patients’ and caregiver partners’ perspectives on several research articles.^67^ The goals of this engagement were to define key advocacy priorities for an IBD patient organization and to highlight content within the report that was most relevant to IBD patients.^67^

In one study by McCormick et al., the involvement of women with breast cancer in environmental causes research shifted the focus of the program from a biochemical model to a political one that better reflected the women’s interests.^68^

PPIn was also cited to enhance the credibility of IBD study tools. This effect has been demonstrated in community and social care research, where evidence suggests public engagement gives both the researchers and the research project greater credibility.^7,69^ For instance, in a research study involving Alaska Natives, community members outperformed researchers in recruiting participants. This was attributed to community members giving legitimacy to claims the project was being conducted in partnership with the community.^70^ Several factors may explain the observed role of PPIn in improving research enrolment. First, as seen in three studies in our cohort, patients may act as active participants in study recruitment, directly supporting the efforts of study authors. Second, patients may indirectly support study recruitment via contributions to research design. Authors of a pediatric RCT on osteopathy attributed the trial’s high enrolment rate to the stakeholders’ contributions to group design.^71^ A RCT on environmental control education in asthma similarly acknowledged the role of a community advisory board in promoting successful recruitment strategies, along with creating study reports that were understandable to the community.^72^

With these benefits in mind, the costs of PPIn, along with recruitment difficulties, may limit the feasibility of incorporating PPIn in IBD research. A literature review by Shah et al. cited time constraints as a key impediment to involving users in the development and evaluation of medical device technologies.^73,74^ PPIn likely represents a significant time commitment for patients and researchers, which may hinder its widespread application. The profile of engaged patients may also differ substantially from that of the general IBD population. Viswanathan et al. (2004) cited a recruitment bias toward highly motivated people in research involvement. In the IBD cohort, such a bias might hamper the applicability of certain quality-of-life interventions, which often require significant investment on the part of the patient.^5^

Study heterogeneity in our sample limits our synthesis of patient engagement in IBD. Best practices of PPIn are likely to vary with the context of engagement. Nevertheless, we outline the following recommendations for implementing participatory research frameworks in IBD. (1) We recommend recruiting engaged parties broadly to ensure findings are generalizable to the IBD population. (2) With time commitment being a key limitation in recruitment, we encourage researchers to elicit regular patient feedback to modify the involvement process in accordance with patient needs. (3) When engaging patients in questionnaire development, we propose employing cognitive interviewing techniques to improve individual item clarity. (4) We recommend engaging patients continuously throughout the research process to address emerging concerns. This is consistent with prior research from Lindenmeyer et al. and Rhodes et al., who found long-term involvement aided user-researcher cohesion and facilitated a deeper knowledge of and contribution to the research process.^69,73^ (5) We recommend authors adhere to reporting guidelines for PPIn such as PORLET and GRIPP2 to better define the scope of PPIn and standardize its methodology.

In addition to the above recommendations, the Canadian Institutes of Health Research has designed a framework highlighting guiding principles, and core areas of patient engagement for adoption by clinical research partners.^75^ The Strategy for Patient-Oriented Research highlights guiding principles for patient engagement such as integrating PPIn into researcher training, implementing tangible incentives, creating a diverse pool of patient participants, and securing financial compensation to support PPIn.^75^

We note several study limitations. Our search likely did not capture all instances of PPIn in IBD, as study authors may not strictly adhere to standardized reporting guidelines defining PPIn such as GRIPP2. In addition, reporting biases may have been present in our sample. The benefits, challenges, and best practices of PPIn in IBD were reported directly by authors and, accordingly, may be subject to several biases, including pre-existing conceptions of the role of PPIn in research.^7^ In the absence of a robust measurement of impact, our interpretation of these findings is limited.^76^ Similar limitations were noted in a 2009 review exploring the impact of public involvement in the NHS, citing a lack of quantitative evidence of benefit, which is largely based on the views of researchers and members of the public.^14^ We were also unable to identify any benefits or challenges reported by engaged patients, who offer a valuable perspective and may represent a hypothesis generating future research ideas. We excluded certain study types (e.g., opinion pieces and editorials), which may feature PPIn. Lastly, the pool of engaged person(s) in our cohort may have experienced selection bias. Due to time availability among other factors, certain IBD patients may be more likely to be recruited.^34^

Considering these limitations, we highlight the implications of our findings. PPIn represents an important step in advancing patient-centred care. Moreover, authors highlighted several recommendations that may be incorporated in future iterations of PPIn in IBD, such as cognitive interviewing and continuous patient involvement.

## Conclusions

PPIn in IBD improved recruitment rates, promoted the creation of user-friendly quality-of-life tools, and ensured patients’ concerns were reflected in the research process. Authors recommended continuous involvement of patients throughout the research process to address their emerging priorities. PPIn in IBD may be limited by recruitment difficulties and selection bias. Future research in this area should focus on assessing the impact of patient engagement on the efficacy of quality-of-life metrics in IBD.

## Supplementary data

Supplementary data are available at *Journal of the Canadian Association of Gastroenterology* online.

gwad054_suppl_Supplementary_Materials

## Data Availability

The data underlying this article are available in the article and in its supplement and where used is cited under References. Additional data may be shared upon request.

## Conference presentations

The present study was presented as a poster in Canadian Digestive Diseases Week 2022 and in Digestive Diseases Week 2023.

## References

[1] Boivin, Antoine, Tessa Richards, Laura Forsythe, Alexandre Grégoire, Audrey L’Espérance, Julia Abelson, Kristin L Carman. “Evaluating Patient and Public Involvement in Research.” BMJ 363, no. 5147 (2018): 1-2. 10.1136/bmj.k5147 30522999

[2] Thornton, Hazel. “Patient and Public Involvement in Clinical Trials.” BMJ 336, no. 7650 (2008): 903–4. 10.1136/bmj.39547.586100.80 18436920 PMC2335270

[3] Gray, Zoë. INVOLVE Supporting Public Involvement in NHS, Public Health and Social Care Research. (2019). http://www.invo.org.uk/about-involve/

[4] About PCORI. PCORI. (2023). https://www.pcori.org/about/about-pcori

[5] Viswanathan Meera , Alice Ammerman, Eugenia Eng, et al. Community-based participatory research: assessing the evidence: summary. In: *AHRQ Evidence Report* Summaries, 1998–2005. 99. Rockville (MD): Agency for Healthcare Research and Quality (US), 2004. PMC4780908 15460504

[6] Brett, Jo, Sophie Staniszewska, Carole Mockford, Sandra Herron-Marx, John Hughes, Colin Tysall, Rashida Suleman. “A Systematic Review of the Impact of Patient and Public Involvement on Service Users, Researchers and Communities.” *Patient* 7, no. 4 (2014): 387–95. 10.1007/s40271-014-0065-0 25034612

[7] Domecq, Juan Pablo, Gabriela Prutsky, Tarig Elraiyah, Zhen Wang, Mohammed Nabhan, Nathan Shippee, Juan Pablo Brito, et al. “Patient Engagement in Research: A Systematic Review.” BMC Health Services Research 14, no. 1 (2014): 89. 10.1186/1472-6963-14-89 24568690 PMC3938901

[8] Barello, Serena, Elena Guida, Salvatore Leone, Enrica Previtali, Guendalina Graffigna. “Does Patient Engagement Affect IBD Patients’ Health-Related Quality of Life? Findings From a Cross-Sectional Study Among People With Inflammatory Bowel Diseases.” Health and Quality of Life Outcomes 19, no. 1 (2021): 1–9. 10.1186/s12955-021-01724-w 33678181 PMC7938585

[9] Wong, Geoff, Trish Greenhalgh, Gill Westhorp, Jeanette Buckingham, Ray Pawson. “Rameses Publication Standards: Meta-Narrative Reviews.” BMC Medicine 11, no. 1 (2013): 21. 10.1186/1741-7015-11-21 23360661 PMC3558334

[10] Tricco, Andrea, Erin Lillie, Wasifa Zarin, Kelly K O’Brien, Heather Colquhoun, Danielle Levac, David Moher, et al. “PRISMA Extension for Scoping Reviews (PRISMA-ScR): Checklist and Explanation.” Annals of Internal Medicine 169, no. 7 (2018): 467–73. 10.7326/M18-0850 30178033

[11] McGowan, Jessie, Margaret Sampson, Douglas Salzwedel, Elise Cogo, Vicki Foerster, Carol Lefebvre. “PRESS Peer Review of Electronic Search Strategies: 2015 Guideline Statement.” Journal of Clinical Epidemiology 2016, no. 75 (2016): 40–6. 10.1016/j.jclinepi.2016.01.021 27005575

[12] Jones, Julia, Marion Cowe, Sue Marks, Tony McAllister, Alex Mendoza, Carole Ponniah, Helena Wythe, Elspeth Mathie. “Reporting on Patient and Public Involvement (PPI) in Research Publications: Using the Gripp2 Checklists with Lay Co-Researchers.” Research Involvement and Engagement 7, no. 1 (2021): 1-13. 10.1186/s40900-021-00295-w 34294148 PMC8296743

[13] Weise, Christina. (2021). PORLET & IRLET. Saskatchewan Centre for Patient-Oriented Research. https://www.scpor.ca/porlet20

[14] Staley, Kristina. (rep.). Exploring Impact: Public Involvement in NHS, Public Health and Social Care Research, 1–116. London, UK: National Health Society, 2009.

[15] Adegbola, Samuel, Lesley Dibley, Kapil Sahnan, Tiffany Wade, Azmina Verjee, Rachel Sawyer, Sameer Mannick, et al. “Development and Initial Psychometric Validation of a Patient-Reported Outcome Measure for Crohn’s Perianal Fistula: The Crohn’s Anal Fistula Quality of Life (CAF-qol) scale.” Gut 70, no. 9 (2020): 1649–56. 10.1136/gutjnl-2019-320553 33272978 PMC8355881

[16] Adegbola, Samuel, Lesley Dibley, Kapil Sahnan, Tiffany Wade, Azmina Verjee, Rachel Sawyer, Sameer Mannick, et al. “Burden of Disease and Adaptation to Life in Patients with Crohn’s Perianal Fistula: A Qualitative Exploration.” Health and Quality of Life Outcomes 18, no. 1 (2020): 1-13. 10.1186/s12955-020-01622-7 33218361 PMC7678264

[17] Adler, Jeremy, Shehzad A Saeed, Ian Eslick, Lloyd Provost, Peter A Margolis, Heather C Kaplan. “Appreciating the Nuance of Daily Symptom Variation to Individualize Patient Care.” eGEMs 4, no. 1 (2016): 8. 10.13063/2327-9214.1247 PMC4909374 27376097

[18] Alnaqbi, Khalid, Zahi Touma, Laura Passalent, Sindhu Johnson, George Tomlinson, Adele Carty, Robert Inman, “Development, Sensibility, and Reliability of the Toronto Axial Spondyloarthritis Questionnaire in Inflammatory Bowel Disease.” Journal of Rheumatology 40, no. 10 (2013): 1726–35. 10.3899/jrheum.130048 23996291

[19] Alrubaiy, Laith, WaiYee Cheung, Phedra Dodds, Hayley Anne Hutchings, Ian Trevor Russell, Alan Watkins, John Gordon Williams. “Development of a Short Questionnaire to Assess the Quality of Life in Crohn’s Disease and Ulcerative Colitis.” Journal of Crohn's and Colitis 9, no. 1 (2014): 66–76. 10.1093/ecco-jcc/jju005 25518049

[20] Bitton, Alain, Katharine Devitt, Brian Bressler, Joan Heatherington, Vipul Jairath, Jennifer Jones, Paul Moayyedi, et al. “Development of a Global Rating Scale for Inflammatory Bowel Sisease.” Journal of the Canadian Association of Gastroenterology 3, no. 1 (2019): 4–16. 10.1093/jcag/gwz017 34169223 PMC8218537

[21] Bitton, Alain, Maria Vutcovici, Ellina Lytvyak, Natasha Kachan, Brian Bressler, Jennifer Jones, Peter Lakatos, et al. “Selection of Quality Indicators in IBD: Integrating Physician and Patient Perspectives.” Inflammatory Bowel Diseases 25, no. 2 (2018): 403–9. 10.1093/ibd/izy259 30169582

[22] Bodger, Keith, Clare Ormerod, Daniela Shackcloth, Melanie Harrison; IBD Control Collaborative. “Development and Validation of a Rapid, Generic Measure of Disease Control From the Patient’s Perspective: The IBD-Control Questionnaire.” Gut 63, no. 7 (2013): 1092–102. 10.1136/gutjnl-2013-305600 24107590 PMC4078750

[23] Carter, Bernie, Alison Rouncefield-Swales, Lucy Bray, Lucy Blake, Stephen Allen, Chris Probert, Kay Crook, Pamela Qualter. ““I Don’t Like to Make a Big Thing Out of iIt”: A Qualitative Interview-Based Study Exploring Factors Affecting Whether Young People Tell or Do Not Tell Their Friends About Their IBD.” International Journal of Chronic Diseases 2020, no. 1 (2020): 1–11. 10.1155/2020/1059025 PMC7305549 32577420

[24] Casellas, Francesc, Daniel Ginard, Isabel Vera, Antonio Torrejón; GETECCU. “Development and Testing of a New Instrument to Measure Patient Satisfaction With Health Care in Inflammatory Bowel Disease.” Inflammatory Bowel Diseases 19, no. 3 (2013): 559–68. 10.1097/MIB.0b013e31827febd1 23429448

[25] Cheifetz, Adam S, Gil Melmed, Brennan Spiegel, Jennifer Talley, Shane Devlin, Laura Raffals, Peter Irving, et al. “Setting Priorities for Comparative Effectiveness Research in Inflammatory Bowel Disease: Results of an International Provider Survey, Expert R and Panel, and Patient Focus Groups.” Inflammatory Bowel Diseases 18, no. 12 (2012): 2294–300. 10.1002/ibd.22920 22337359

[26] de Jong, Marin, Andrea van der Meulen-de Jong, Mariëlle Romberg-Camps, Juliette Degens, Marco Becx, Tineke Markus, Henny Tomlow, et al. “Development and Feasibility Study of a Telemedicine Tool for All Patients with IBD.” Inflammatory Bowel Diseases 23, no. 4 (2017): 485–93. 10.1097/MIB.0000000000001034 28267047

[27] Denters Maaike , Marije Deutekom, Bert Derkx, Patrick Bossuyt, Paul Fockens, Evelien Dekker. “Patient Satisfaction with the Colonoscopy Procedure: Endoscopists Overestimate the Importance of Adverse Physical Symptoms.” Frontline Gastroenterology 3, no. 3 (2012): 130–6. 10.1136/flgastro-2012-100150 28839653 PMC5517283

[28] Dibley, Lesley, Wladyslawa Czuber-Dochan, Sue Woodward, Tiffany Wade, Paul Bassett, Jackie Sturt, Christine Norton, et al. “Development and Psychometric Properties of the Inflammatory Bowel Disease Distress Scale (IBD-DS): A New Tool to Measure Disease-Specific Distress.” Inflammatory Bowel Diseases 24, no. 9 (2018): 2068–77. 10.1093/ibd/izy108 29788323

[29] Dibley, Lesley, Bernadette Khoshaba, Micol Artom, Victoria Van Loo, Louise Sweeney, Jonathan Syred, Sula Windgassen, Georgia Moffatt, Christine Norton; members of the IBD-BOOST PPI team. “Patient Strategies for Managing the Vicious Cycle of Fatigue, Pain and Urgency in Inflammatory Bowel Disease: Impact, Planning and Support.” Digestive Diseases and Sciences 66, no. 10 (2020): 3330–42. 10.1007/s10620-020-06698-1 33164146

[30] Fofaria, Rishi, Susan Barber, Yewande Adeleke, Tom Woodcock, Nikolaos Kamperidis, Alaa Mohamed, Ravi Misra, et al. “Stratification of Inflammatory Bowel Disease Outpatients by Disease Activity and Risk of Complications to Guide Out-of-Hospital Monitoring: A Patient-Centred Quality Improvement Project.” BMJ Open Quality 8, no. 3 (2019): e000546. 10.1136/bmjoq-2018-000546 PMC6683110 31428704

[31] Grant, Amy, Melissa Crane, Andreas Laupacis, Anne Griffiths, Dave Burnett, Amanda Hood, Cheryl Kluthe, et al. “Engaging Patients and Caregivers in Research for Pediatric Inflammatory Bowel Disease: Top 10 Research Priorities.” Journal of Pediatric Gastroenterology and Nutrition 69, no. 3 (2019): 317–23. 10.1097/MPG.0000000000002396 31436670

[32] Guida, Laura, Francesca Maria Di Giorgio, Anita Busacca, Lucio Carrozza, Stefania Ciminnisi, PL. Almasio, Vito Di Marco, Maria Cappello. “Perception of the Role of Food and Dietary Modifications in Patients with Inflammatory Bowel Disease: Impact on Lifestyle.” Nutrients 13, no. 3 (2021): 759. 10.3390/nu13030759 33652848 PMC7996868

[33] Haaland, Derek, Andrew Day, Anthony Otley. “Development and Validation of a Pediatric IBD Knowledge Inventory Device.” Journal of Pediatric Gastroenterology and Nutrition 58, no. 3 (2014): 313–9. 10.1097/MPG.0000000000000210 24135980

[34] Hart, Alisa, Miranda Lomer, Azmina Verjee, Karen Kemp, Omar Faiz, Ann Daly, Julie Solomon, John McLaughlin. “What Are the Top 10 Research Questions in the Treatment of Inflammatory Bowel Disease? A Priority Setting Partnership With the James Lind Alliance.” Journal of Crohn’s and Colitis 11, no. 2 (2016): 204–11. 10.1093/ecco-jcc/jjw144 PMC5266081 27506537

[35] Hubbard, Gill, Rebecca Beeken, Claire Taylor, Angus Watson, Julie Munro, William Goodman. “A Physical Activity Intervention to Improve the Quality of Life of Patients With a Stoma: A Feasibility Study Protocol.” Pilot and Feasibility Studies 5, no. 1 (2019): 78. 10.1186/s40814-019-0461-2 31236285 PMC6580610

[36] Hughes, Lyndsay, Laura King, Myfanwy Morgan, Salma Ayis, Natalie Direkze, Miranda Lomer, James Lindsay, Kevin Whelan. “Food-Related Quality of Life in Inflammatory Bowel Disease: Development and Validation of a Questionnaire.” Journal of Crohn's and Colitis 10, no. 2 (2015): 194–201. 10.1093/ecco-jcc/jjv192 26507859

[37] Kapasi, Rukshana, Jackie Glatter, Christopher Lamb, Austin Acheson, Charles Andrews, Ian Arnott, Kevin Barrett, et al. “Consensus Standards of Healthcare for Adults and Children with Inflammatory Bowel Disease in the UK.” Frontline Gastroenterology 11, no. 3 (2019): 178–87. 10.1136/flgastro-2019-101260 32419908 PMC7223296

[38] Katarina, Pihl Lesnovska, Börjeson Sussanne, Hollman Frisman Gunilla, Hjortswang Henrik, Wenemark Marika. “The Quality of Care Questionnaire: Development of a Valid Measure for Persons With Inflammatory Bowel Disease.” Scandinavian Journal of Gastroenterology 53, no. 9 (2018): 1043–50. 10.1080/00365521.2018.1495759 30299173

[39] Kennedy, Anne, E. Andrea Nelson, David Reeves, Gerald Richardson, Chris Roberts, A Robinson, Anne Rogers, Mark Sculpher, David Thompson. “A Randomised Controlled Trial to Assess the Impact of a Package Comprising a Patient-Orientated, Evidence-Based Self-Help Guidebook and Patient-Centred Consultations on Disease Management and Satisfaction in Inflammatory Bowel Disease.” Health Technology Assessment 7, no. 28 (2003): iii, 1–113. 10.3310/hta7280 14567905

[40] Kennedy, Anne, Anne Rogers. “Improving Patient Involvement in Chronic Disease Management: The Views of Patients, GPS and Specialists on a Guidebook for Ulcerative Colitis.” Patient Education and Counseling 47, no. 3 (2002): 257–63. 10.1016/s0738-3991(01)00228-2 12088604

[41] Kennedy, Samantha, Michele Herzer Maddux. “Patient–Clinician Collaboration in the Development of an IBD Transfer Toolkit.” Pediatrics 144, no. 3 (2009): 257-263. 10.1542/peds.2019-0558 31391212

[42] Khalil, Carine, Welmoed Van Deen, Taylor Dupuy, Nirupama Bonthala, Christopher Almario, Brennan Spiegel. “Developing Patient-Centered Inflammatory Bowel Disease–Related Educational Videos Optimized for Social Media: Qualitative Research Study.” JMIR Medical Education 6, no. 2 (2020): e21639. 10.2196/21639 33079065 PMC7609199

[43] Kim, Andrew, Afaf Girgis, Peter De Cruz, Corey Siegel, Neda Karimi, Sasha Ruban, Alexandra Sechi, Wa Sang Watson Ng, Jane Andrews, Susan Connor. “Development and Feasibility of a Web-Based Decision Aid for Patients With Ulcerative Colitis: Qualitative Pilot Study.” Journal of Medical Internet Research 23, no. 2 (2021): 1-17. 10.2196/15946 PMC7952232 33629956

[44] Kim, Andrew, Charlotte Roberts, Brian Feagan, Rupa Banerjee, Willem Bemelman, Keith Bodger, Marc Derieppe, et al. “Developing a Standard Set of Patient-Centred Outcomes for Inflammatory Bowel Disease—An International, Cross-Disciplinary Consensus.” Journal of Crohn’s and Colitis 12, no. 4 (2017): 408–18. 10.1093/ecco-jcc/jjx161 29216349

[45] Lee, Matthew, Georgina Jones, Alan Lobo, Steven Brown, Robert Bethune, Alex Blackmore, Nicole Fearnhead, et al. “Survey to define informational needs of patients undergoing surgery for crohn’s anal fistula.” Colorectal Disease 23, no. 1 (2020): 132–44. 10.1111/codi.15423 33140914

[46] Louis, Edouard, Juan Manuel Ramos-Goñi, Jesus Cuervo, Uri Kopylov, Manuel Barreiro-de Acosta, Sara McCartney, Greg Rosenfeld, et al. “A Qualitative Research for Defining Meaningful Attributes for the Treatment of Inflammatory Bowel Disease From the Patient Perspective.” Patient 13, no. 3 (2020): 317–25. 10.1007/s40271-019-00407-5 31997116 PMC7210247

[47] Lovén Wickman, Ulrica, Pia Yngman-Uhlin, Henrik Hjortswang, Marika Wenemark, Henrik Stjernman, Barbara Riegel, Gunilla Hollman Frisman. “Development of a Self-Care Questionnaire for Clinical Assessment of Self-Care in Patients with Inflammatory Bowel Disease: A Psychometric Evaluation.” International Journal of Nursing Studies 89, no. 1 (2019): 1–7. 10.1016/j.ijnurstu.2018.08.016 30316054

[48] Macdonald, Graham George, Cheryl Koehn, Gail Attara, Allan Stordy, Marilee Allerdings, Jenny Leese, Linda Li, Catherine Backman. “Patient Perspectives on the Challenges and Responsibilities of Living with Chronic Inflammatory Diseases: Qualitative Study.” Jouranl of Participatory Medicine 10, no. 4 (2018): e10815. 10.2196/10815 PMC7434083 33052129

[49] Marín-Jiménez, Ignacio, Milena Gobbo Montoya, Abel Panadero, Mercedes Cañas, Yolanda Modino, Cristina Romero de Santos, Jordi Guardiola, Loreto Carmona, Manuel Barreiro-de Acosta. “Management of the Psychological Impact of Inflammatory Bowel Disease.” Inflammatory Bowel Diseases 23, no. 9 (2017): 1492–8. 10.1097/MIB.0000000000001205 28786866

[50] McDermott, Edel, Gerard Healy, Georgina Mullen, Denise Keegan, Kathryn Byrne, Allys Guerandel, Mary Forry, et al. “Patient Education in Inflammatory Bowel Disease: A Patient-Centred, Mixed Methodology Study.” Journal of Crohn's and Colitis 12, no. 4 (2017): 419–24. 10.1093/ecco-jcc/jjx175 29293956

[51] Oliver, Brant, Alice Kennedy, Welmoed van Deen, Alandra Weaver, Caren Heller, Megan Holthoff, Jeffrey Bank, Gil Melmed, Corey Siegel, Eugene Nelson. “Development of Balanced Whole System Value Measures for Inflammatory Bowel Disease Care in the IBD Qorus Collaborative Using a Modified Delphi Process.” Inflammatory Bowel Diseases 28, no. 3 (2021): 327–36. 10.1093/ibd/izab091 34037211

[52] Rohatinsky, Noelle, Ian Boyd, Alyssa Dickson, Sharyle Fowler, Juan-Nicolás Peña-Sánchez, Carol-Lynne Quintin, Tracie Risling, Brooke Russell, Kendall Wicks, Mike Wicks. “Perspectives of Health Care Use and Access to Care for Individuals Living with Inflammatory Bowel Disease in Rural Canada.” Rural and Remote Health 21, no. 2 (2021): 1-11. 10.22605/rrh6358 33820422

[53] Ruan, Jiayin, Yan Chen, Yunxian Zhou. “Development and Validation of a Questionnaire to Assess the Quality of Life in Patients with Inflammatory Bowel Disease in Mainland China.” Inflammatory Bowel Diseases 23, no. 3 (2017): 431–9. 10.1097/MIB.0000000000001024 28129287

[54] Vent-Schmidt, Jens, Laurie Goldsmith, Theodore Steiner. “Patients’ Willingness and Perspectives Toward Chimeric Antigen Receptor T-Regulatory Cell Therapy for Inflammatory Bowel Diseases.” Crohn’s Colitis 360 2, no. 4 (2020): otaa085. 10.1093/crocol/otaa085 36777762 PMC9802168

[55] Vergara, Mercè, Francesc Casellas, Xavier Badia, Juan Malagelada. “Assessing the Quality of Life of Household Members of Patients with Inflammatory Bowel Disease: Development and Validation of a Specific Questionnaire.” American Journal of Gastroenterology 97, no. 6 (2002): 1429–37. 10.1111/j.1572-0241.2002.05684.x 12094861

[56] Williams, Astrid-Jane, Neda Karimi, Radha Chari, Susan Connor, Mary De Vera, Levinus Dieleman, Tawnya Hansen, et al. “Shared Decision Making in Pregnancy in Inflammatory Bowel Disease: Design of a Patient Orientated Decision Aid.” BMC Gastroenterology 21, no. 1 (2020): 1-18. 10.21203/rs.3.rs-131067/v1 PMC8325254 34330215

[57] Sahnan, Kapil, Phil Tozer, Samuel Adegbola, Matthew Lee, Nick Heywood, Angus McNair, Daniel Hind, et al. “Developing a Core Outcome Set for Fistulising Perianal Crohn’s Disease.” Gut 68, no. 2 (2018): 226–38. 10.1136/gutjnl-2017-315503 29437911 PMC6352412

[58] Heisler, Courtney, Noelle Rohatinsky, Raza Mirza, Olga Kits, Sandra Zelinsky, Sander Veldhuyzen van Zanten, Geoffrey Nguyen, et al. “Patient-Centered Access to IBD Care: A Qualitative Study.” Crohn’s & Colitis 360 5, no. 1 (2022): otac045. 10.1093/crocol/otac045 PMC9825304 36777367

[59] Santos, José Diego Marques, Sharyle Fowler, Derek Jennings, Colten Brass, Linda Porter, Robert Porter, Rhonda Sanderson, Juan Nicolás Peña-Sánchez. “Health Care Utilization Differences Between First Nations People and the General Population with Inflammatory Bowel Disease: A Retrospective Cohort Study From Saskatchewan, Canada.” CMAJ Open 10, no. 4 (2022): E964-E970. 10.9778/cmajo.20220118 PMC9633056 36319027

[60] Cavallaro, Paul, Nicola Fearnhead, Ian Bissett, Mantaj Brar, Thomas Cataldo, Rasheed Clarke, Paula Denoya, et al.; On behalf of the PROPS Delphi Study Expert Panels. “Patients Undergoing Ileoanal Pouch Surgery Experience a Constellation of Symptoms and Consequences Representing a Unique Syndrome.” Annals of Surgery 274, no. 1 (2021): 138–45. 10.1097/SLA.0000000000004829 33914449 PMC8968673

[61] Rines, Jenna, Kim Daley, Sunny Loo, Kwestan Safari, Deirdre Walsh, Marlyn Gill, Paul Moayyedi, Aida Fernandes, Nancy Marlett, Deborah Marshall. “A Patient-Led, Peer-to-Peer Qualitative Study on the Psychosocial Relationship Between Young Adults With Inflammatory Bowel Disease and Food.” Health Expectations 25, no. 4 (2022): 1486–97. 10.1111/hex.13488 35383400 PMC9327832

[62] Long, Millie, Welmoed van Deen, Laura Weisbein, Carine Khalil, Keren Appel, Xian Zhang, Wenli Chen, et al. “Web-Based Video Education to Improve Uptake of Influenza Vaccination and Other Preventive Health Recommendations in Adults with Inflammatory Bowel Disease: Randomized Controlled Trial of Project Prevent.” Journal of Medical Internet Research 25, no. 1 (2023): 1-12. 10.2196/42921 PMC10483303 37610821

[63] Gorbenko, Ksenia, Alexa Rae Riggs, Brooke Koeppel, Sydney Phlegar, Marla Dubinsky, Ryan Ungaro, Laurie Keefer. “Photovoice as a Tool to Improve Patient—Provider Communication in Inflammatory Bowel Disease Clinic: A Feasibility Study.” Journal of Evaluation in Clinical Practice 28, no. 1 (2021): 159–68. 10.1111/jep.13609 34382292

[64] Peña-Sánchez, Juan Nicolás, Jessica Amankwah Osei, Jose Diego Marques Santos, Derek Jennings, Mustafa Andkhoie, Colten Brass, Germain Bukassa-Kazadi, et al. “Increasing Prevalence and Stable Incidence Rates of Inflammatory Bowel Disease Among First Nations: Population-Based Evidence From A Western Canadian Province.” Inflammatory Bowel Diseases 28, no. 4 (2021): 514–22. 10.1093/ibd/izab096 PMC8972279 34037223

[65] Peña-Sánchez, Juan Nicolás, Jessica Amankwah Osei, Noelle Rohatinsky, Xinya Lu, Tracie Risling, Ian Boyd, Kendall Wicks, et al. “Inequities in Rural and Urban Health Care Utilization Among Individuals Diagnosed with Inflammatory Bowel Disease: A Retrospective Population-Based Cohort Study from Saskatchewan, Canada.” Journal of the Canadian Association of Gastroenterology 6, no. 2 (2022): 55–63. 10.1093/jcag/gwac015 37025513 PMC10071297

[66] Bryant, Louise, Judith Beckett, Allan House, Cath Sweeney. (rep.). The Practicality and Acceptability of an Advocacy Service in the Emergency Department for People Attending Following Self-Harm. 1–134. Leeds, UK: University of Leeds, 2006.

[67] Windsor, Joseph, M Ellen Kuenzig, Sanjay Murthy, Alain Bitton, Charles Bernstein, Jennifer Jones, Kate Lee, et al. “The 2023 Impact of Inflammatory Bowel Disease in Canada: Executive Summary.” Journal of the Canadian Association of Gastroenterology 6, no. Supplement_2 (2023): S1-S8. 10.1093/jcag/gwad003 37674500 PMC10478799

[68] McCormick, Sabrina, Julia Brody, Phil Brown, Ruth Polk. “Public Involvement in Breast Cancer Research: An Analysis and Model for Future Research.” International Journal of Health Services 34, no. 4 (2004): 625–46. 10.2190/HPXB-9RK8-ETVM-RVEA 15560426

[69] Rhodes, Penny, Andrew Nocon, Michael Booth, MY Chowdrey, Anne Fabian, Neville Lambert, Faqir Mohammed, Teresa Walgrove. “A Service Users’ Research Advisory Group From the Perspectives of Both Service Users and Researchers.” Health and Social Care in the Community 10, no. 5 (2002): 402–9. 10.1046/j.1365-2524.2002.00376.x 12390226

[70] Allen, James, Gerald Mohatt, S Michelle Rasmus, Kelly Hazel, Lisa Thomas, Sharon Lindley. “The Tools to Understand: Community as Co-Researcher on Culture-Specific Protective Factors for Alaska Natives.” Journal of Prevention & Intervention in the Community 32, no. 1-2 (2006): 41–59. 17000601 10.1300/J005v32n01_04

[71] Edwards, Vanessa, Katrina Wyatt, Stuart Logan, Nicky Britten. “Consulting Parents About the Design of a Randomized Controlled Trial of Osteopathy for Children with Cerebral Palsy.” Health Expectations 14, no. 4 (2011): 429–38. 10.1111/j.1369-7625.2010.00652.x 21244590 PMC5060591

[72] Swartz, Lee J, Karen A Callahan, Arlene M Butz, Cynthia S Rand, Sukon Kanchanaraksa, Gregory B Diette, Jerry A Krishnan, et al. “Methods and Issues in Conducting a Community-Based Environmental Randomized Trial.” Environmental Research 95, no. 2 (2004): 156–65. 10.1016/j.envres.2003.08.003 15147921

[73] Lindenmeyer, Antje, Hilary Hearnshaw, Jackie Sturt, Ralph Ormerod, Geoff Aitchison. “Assessment of the Benefits of User Involvement in Health Research from the Warwick Diabetes Care Research User Group: A Qualitative Case Study.” Health Expectations 10, no. 3 (2007): 268–77. 10.1111/j.1369-7625.2007.00451.x 17678515 PMC5060408

[74] Shah, Syed Ghulam Sarwar, Ian Robinson. “Benefits of and Barriers to Involving Users in Medical Device Technology Development and Evaluation.” International Journal of Technology Assessment in Health Care 23, no. 1 (2007): 131–7. 10.1017/S0266462307051677 17234027

[75] Government of Canada, Canadian Institutes of Health Research (2019, May 27). Strategy for Patient-Oriented Research—Patient Engagement Framework. CIHR. https://cihr-irsc.gc.ca/e/48413.html

[76] Mockford, Carole, Sophie Staniszewska, Frances Griffiths, Sandra Herron-Marx. “The Impact of Patient and Public Involvement on UK NHS Health Care: A Systematic Review.” International Journal for Quality in Health Care 24, no. 1 (2011): 28–38. 10.1093/intqhc/mzr066 22109631

