# Supplementary material for: Patient and Public Involvement in Inflammatory Bowel Disease Research—A Scoping Review
Source: J Can Assoc Gastroenterol. 2023 Dec 14;7(2):137–48. doi: 10.1093/jcag/gwad054 (PMC10999768; doi:10.1093/jcag/gwad054)
Supplement: gwad054_suppl_Supplementary_Materials [file gwad054_suppl_supplementary_materials.zip › gwad054_suppl_Supplementary_Content_2.docx]

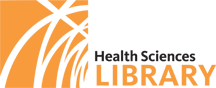


**Patient and public involvement (PPI) in IBD research – a systematic review UPDATE**

**Requestor(s) name and email**:

Karam Elsolh

[karam.elsolh@medportal.ca](mailto:karam.elsolh@medportal.ca)

**Principle Investigator name and email:**

Dr. Samir Grover

[Samir.Grover@unityhealth.to](mailto:Samir.Grover@unityhealth.to)

**Date:** August 21, 2023

**Information Specialist**:

Teruko Kishibe

Archivist/Information Specialist

Health Sciences Library, St. Michael's

30 Bond Street, Toronto, Ontario M5B 1W8

[Teruko.Kishibe@unityhealth.to](mailto:Teruko.Kishibe@unityhealth.to)

Table of Contents

[Research Question 2](#_Toc143516522)

[Description 2](#_Toc143516523)

[Limits 2](#_Toc143516524)

[Number of results per database 2](#_Toc143516525)

[Search Strategies 3](#_Toc143516526)

[Medline 3](#_Toc143516527)

[Cochrane Central 4](#_Toc143516528)

[Embase 5](#_Toc143516529)

[Results: Titles & Abstracts 6](#_Toc143516530)

# Research Question

What are the benefits, costs, challenges, recruitment & engagement strategies, and best practices in PPI among IBD research?

## Description

The search strategy takes the general format of Inflammatory Bowel Disease AND Patient Involvement. This is an update to a search originally run on September 8, 2021.

## Limits

Entry date limits have been added to the original search strategies to capture references added to the databases since September 8, 2021.

# Number of results per database

| **Databases Searched** | **Date of Search** | **Number of Results** |
| --- | --- | --- |
| All Ovid Medline <1946 - present> | August 21, 2023 | 662 |
| EBM Reviews - Cochrane Central Register of Controlled Trials <July 2023> | August 21, 2023 | 86 |
| Embase Classic+Embase <1947 to 2023 August 18> | August 21, 2023 | 836 |
| Total | 1584 |  |
| **Total**  **(After deduplication)** | **1163** |  |

# Search Strategies

## Medline

1 exp Inflammatory Bowel Diseases/ 97344

2 polyps/ or exp intestinal polyps/ 27575

3 exp Adenomatous Polyps/ 8756

4 exp Colonoscopy/ 35052

5 (inflamm* adj2 (bowel or colon*)).tw,kf. 69447

6 IBD.tw,kf. 35647

7 (colitide* or colitis or colorectitis or Crohn* or enterocolitis or enteritis or ileitides or ileitis or ileocolitis or pancolitis or proctitis or proctosigmoiditis or rectocolitis or rectosigmoiditis or sigmoiditis).tw,kf. 145823

8 (polyp or polyps or adenoma*).tw,kf. 128383

9 ((adenomatous or famil* or hereditary or inherit*) adj3 polyposis).tw,kf. 10796

10 (colonoscop* or coloscop* or sigmoidoscop* or chromoscop*).tw,kf. 40766

11 1 or 2 or 3 or 4 or 5 or 6 or 7 or 8 or 9 or 10 355720

12 exp Community Participation/ 48019

13 exp Patient-Centered Care/ 24215

14 *Patient Selection/ 18756

15 *patient reported outcome measures/ 5618

16 *Caregivers/ 32208

17 *Focus Groups/ 1397

18 *"Surveys and Questionnaires"/ 50045

19 ((patient* or carer* or caregiver* or family or surrogate* or parent* or communit* or consumer* or stakeholder* or client* or citizen* or public or advocat*) adj5 (involvement or partner* or engage* or empowerment or advocacy or representation* or consult* or perspective* or centered or centred or participation or collaborat* or voice* or unvoiced or narrative* or opinion* or dialog* or input*)).tw,kf. 329214

20 12 or 13 or 14 or 15 or 16 or 17 or 18 or 19 466720

21 11 and 20 4091

22 (202109* or 202110* or 202111* or 202112* or 2022* or 2023*).dt,ez,da. 3669037

23 21 and 22 659

24 limit 21 to ed=20210908-20230821 439

25 23 or 24 662

<https://ovidsp.ovid.com/ovidweb.cgi?T=JS&NEWS=N&PAGE=main&SHAREDSEARCHID=2j3qmCcnw0to1x5SQ5GAqtReOwGFsdeUEoD1CulIrAfDeosDHk0eMc0llXbFQ7Iub>

## Cochrane Central

1 exp Inflammatory Bowel Diseases/ 4733

2 polyps/ or exp intestinal polyps/ 1006

3 exp Adenomatous Polyps/ 291

4 exp Colonoscopy/ 3086

5 (inflamm* adj2 (bowel or colon*)).tw. 3818

6 IBD.tw. 2292

7 (colitide* or colitis or colorectitis or Crohn* or enterocolitis or enteritis or ileitides or ileitis or ileocolitis or pancolitis or proctitis or proctosigmoiditis or rectocolitis or rectosigmoiditis or sigmoiditis).tw. 13009

8 (polyp or polyps or adenoma*).tw. 8956

9 ((adenomatous or famil* or hereditary or inherit*) adj3 polyposis).tw. 283

10 (colonoscop* or coloscop* or sigmoidoscop* or chromoscop*).tw. 8575

11 1 or 2 or 3 or 4 or 5 or 6 or 7 or 8 or 9 or 10 28916

12 exp Consumer Participation/ 2425

13 exp Patient-Centered Care/ 1035

14 *Patient Selection/ 0

15 *patient reported outcome measures/ 0

16 *Caregivers/ 0

17 *Focus Groups/ 0

18 *"Surveys and Questionnaires"/ 0

19 ((patient* or carer* or caregiver* or family or surrogate* or parent* or communit* or consumer* or stakeholder* or client* or citizen* or public or advocat*) adj5 (involvement or partner* or engage* or empowerment or advocacy or representation* or consult* or perspective* or centered or centred or participation or collaborat* or voice* or unvoiced or narrative* or opinion* or dialog* or input*)).tw. 35258

20 12 or 13 or 14 or 15 or 16 or 17 or 18 or 19 37188

21 11 and 20 574

22 (2021-09* or 2021-10* or 2021-11* or 2021-12* or 2022* or 2023*).dl. 261139

23 21 and 22 86

<https://ovidsp.ovid.com/ovidweb.cgi?T=JS&NEWS=N&PAGE=main&SHAREDSEARCHID=3y0NuWeZgG7nOcnpG7Y0aJHfaZZ9phOQABP7OyrxUxXsUDmTyZ3jwuHOYnZT5yKgj>

## Embase

1 exp inflammatory bowel disease/ 204561

2 polyp/ 26523

3 exp intestine polyp/ 40982

4 adenomatous polyp/ 10661

5 exp colonoscopy/ 104232

6 (inflamm* adj2 (bowel or colon*)).tw,kw. 112099

7 IBD.tw,kw. 73409

8 (colitide* or colitis or colorectitis or Crohn* or enterocolitis or enteritis or ileitides or ileitis or ileocolitis or pancolitis or proctitis or proctosigmoiditis or rectocolitis or rectosigmoiditis or sigmoiditis).tw,kw. 234188

9 (polyp or polyps or adenoma*).tw,kw. 191270

10 ((adenomatous or famil* or hereditary or inherit*) adj3 polyposis).tw,kw. 14379

11 (colonoscop* or coloscop* or sigmoidoscop* or chromoscop*).tw,kw. 81966

12 1 or 2 or 3 or 4 or 5 or 6 or 7 or 8 or 9 or 10 or 11 585812

13 patient participation/ 35588

14 exp community participation/ 4862

15 *patient selection/ 10664

16 *patient-reported outcome/ 16426

17 *caregiver/ 30302

18 *information processing/ 42548

19 exp *questionnaire/ 45239

20 ((patient* or carer* or caregiver* or family or surrogate* or parent* or communit* or consumer* or stakeholder* or client* or citizen* or public or advocat*) adj5 (involvement or partner* or engage* or empowerment or advocacy or representation* or consult* or perspective* or centered or centred or participation or collaborat* or voice* or unvoiced or narrative* or opinion* or dialog* or input*)).tw,kw. 471375

21 13 or 14 or 15 or 16 or 17 or 18 or 19 or 20 627480

22 12 and 21 9220

23 limit 22 to embase 4186

24 limit 23 to dc=20210908-20230821 836

<https://myaccess.library.utoronto.ca/login?url=http://ovidsp.ovid.com/ovidweb.cgi?T=JS&NEWS=N&PAGE=main&SHAREDSEARCHID=6ZUnTdW4azAFPOyTrsIshS7T5DNzgwretqCdA6IZuQUeVZfLir1ykn9zHvz6IGxgD>

# Results: Titles & Abstracts

Please see attached compressed EndNote library and RIS file.


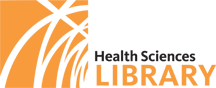


**Patient and public involvement (PPI) in IBD research – a systematic review**

**Requestor(s) name and email**:

Karam Elsolh

[karam.elsolh@medportal.ca](mailto:karam.elsolh@medportal.ca)

**Principle Investigator name and email:**

Dr. Samir Grover

[Samir.Grover@unityhealth.to](mailto:Samir.Grover@unityhealth.to)

**Date:** September 10, 2021

**Information Specialist**:

Teruko Kishibe

Archivist/Information Specialist

Health Sciences Library, St. Michael's

30 Bond Street, Toronto, Ontario M5B 1W8

[Teruko.Kishibe@unityhealth.to](mailto:Teruko.Kishibe@unityhealth.to)

Table of Contents

[Research Question 2](#_Toc82175899)

[Description 2](#_Toc82175900)

[Limits 2](#_Toc82175901)

[Number of results per database 2](#_Toc82175902)

[Search Strategies 3](#_Toc82175903)

[Medline 3](#_Toc82175904)

[Cochrane Central 4](#_Toc82175905)

[Embase 5](#_Toc82175906)

[Results: Titles & Abstracts 5](#_Toc82175907)

# Research Question

What are the benefits, costs, challenges, recruitment & engagement strategies, and best practices in PPI among IBD research?

## Description

The search strategy takes the general format of Inflammatory Bowel Disease AND Patient Involvement.

## Limits

N/A

# Number of results per database

| **Databases Searched** | **Date of Search** | **Number of Results** |
| --- | --- | --- |
| All Ovid Medline <1946 - present> | September 8, 2021 | 3574 |
| EBM Reviews - Cochrane Central Register of Controlled Trials <August 2021> | September 8, 2021 | 674 |
| Embase Classic+Embase <1947 to 2021 September 07> | September 8, 2021 | 3523 |
| Total | 7771 |  |
| **Total**  **(After deduplication)** | 5362 |  |

# Search Strategies

## Medline

1 exp Inflammatory Bowel Diseases/ 86558

2 polyps/ or exp intestinal polyps/ 26053

3 exp Adenomatous Polyps/ 8289

4 exp Colonoscopy/ 32130

5 (inflamm* adj2 (bowel or colon*)).tw,kf. 58608

6 IBD.tw,kf. 29045

7 (colitide* or colitis or colorectitis or Crohn* or enterocolitis or enteritis or ileitides or ileitis or ileocolitis or pancolitis or proctitis or proctosigmoiditis or rectocolitis or rectosigmoiditis or sigmoiditis).tw,kf. 130224

8 (polyp or polyps or adenoma*).tw,kf. 118883

9 ((adenomatous or famil* or hereditary or inherit*) adj3 polyposis).tw,kf. 10252

10 (colonoscop* or coloscop* or sigmoidoscop* or chromoscop*).tw,kf. 36327

11 1 or 2 or 3 or 4 or 5 or 6 or 7 or 8 or 9 or 10 322458

12 exp Community Participation/ 45194

13 exp Patient-Centered Care/ 22114

14 *Patient Selection/ 18362

15 *patient reported outcome measures/ 4695

16 *Caregivers/ 28342

17 *Focus Groups/ 1379

18 *"Surveys and Questionnaires"/ 48969

19 ((patient* or carer* or caregiver* or family or surrogate* or parent* or communit* or consumer* or stakeholder* or client* or citizen* or public or advocat*) adj5 (involvement or partner* or engage* or empowerment or advocacy or representation* or consult* or perspective* or centered or centred or participation or collaborat* or voice* or unvoiced or narrative* or opinion* or dialog* or input*)).tw,kf. 273884

20 12 or 13 or 14 or 15 or 16 or 17 or 18 or 19 405072

21 11 and 20 3574

<https://ovidsp.ovid.com/ovidweb.cgi?T=JS&NEWS=N&PAGE=main&SHAREDSEARCHID=15VKjtusVWcomgE7sA1VCWdoJzTC2BkZF5eUft5bwxjgPz0PoNRdXBDotfoOIkhx2>

## Cochrane Central

1 exp Inflammatory Bowel Diseases/ 3469

2 polyps/ or exp intestinal polyps/ 766

3 exp Adenomatous Polyps/ 251

4 exp Colonoscopy/ 2155

5 (inflamm* adj2 (bowel or colon*)).tw. 3800

6 IBD.tw. 2276

7 (colitide* or colitis or colorectitis or Crohn* or enterocolitis or enteritis or ileitides or ileitis or ileocolitis or pancolitis or proctitis or proctosigmoiditis or rectocolitis or rectosigmoiditis or sigmoiditis).tw. 12096

8 (polyp or polyps or adenoma*).tw. 8255

9 ((adenomatous or famil* or hereditary or inherit*) adj3 polyposis).tw. 287

10 (colonoscop* or coloscop* or sigmoidoscop* or chromoscop*).tw. 7775

11 1 or 2 or 3 or 4 or 5 or 6 or 7 or 8 or 9 or 10 26648

12 exp Consumer Participation/ 1456

13 exp Patient-Centered Care/ 787

14 *Patient Selection/ 0

15 *patient reported outcome measures/ 0

16 *Caregivers/ 0

17 *Focus Groups/ 0

18 *"Surveys and Questionnaires"/ 0

19 ((patient* or carer* or caregiver* or family or surrogate* or parent* or communit* or consumer* or stakeholder* or client* or citizen* or public or advocat*) adj5 (involvement or partner* or engage* or empowerment or advocacy or representation* or consult* or perspective* or centered or centred or participation or collaborat* or voice* or unvoiced or narrative* or opinion* or dialog* or input*)).tw. 39406

20 12 or 13 or 14 or 15 or 16 or 17 or 18 or 19 40614

21 11 and 20 674

<https://ovidsp.ovid.com/ovidweb.cgi?T=JS&NEWS=N&PAGE=main&SHAREDSEARCHID=6DOBPHUxrQ2pCNKFNFpEwc00WF15CDTRYN7FbW9zTbYfQzDLrD05kuvFesz2YXyNM>

## Embase

1 exp inflammatory bowel disease/ 173472

2 polyp/ 24575

3 exp intestine polyp/ 35857

4 adenomatous polyp/ 9883

5 exp colonoscopy/ 88191

6 (inflamm* adj2 (bowel or colon*)).tw,kw. 98320

7 IBD.tw,kw. 59978

8 (colitide* or colitis or colorectitis or Crohn* or enterocolitis or enteritis or ileitides or ileitis or ileocolitis or pancolitis or proctitis or proctosigmoiditis or rectocolitis or rectosigmoiditis or sigmoiditis).tw,kw. 207486

9 (polyp or polyps or adenoma*).tw,kw. 179247

10 ((adenomatous or famil* or hereditary or inherit*) adj3 polyposis).tw,kw. 13829

11 (colonoscop* or coloscop* or sigmoidoscop* or chromoscop*).tw,kw. 71137

12 1 or 2 or 3 or 4 or 5 or 6 or 7 or 8 or 9 or 10 or 11 521543

13 patient participation/ 30080

14 exp community participation/ 3403

15 *patient selection/ 10007

16 *patient-reported outcome/ 11229

17 *caregiver/ 24831

18 *information processing/ 40527

19 exp *questionnaire/ 41505

20 ((patient* or carer* or caregiver* or family or surrogate* or parent* or communit* or consumer* or stakeholder* or client* or citizen* or public or advocat*) adj5 (involvement or partner* or engage* or empowerment or advocacy or representation* or consult* or perspective* or centered or centred or participation or collaborat* or voice* or unvoiced or narrative* or opinion* or dialog* or input*)).tw,kw. 400558

21 13 or 14 or 15 or 16 or 17 or 18 or 19 or 20 537571

22 12 and 21 7478

23 limit 22 to embase 3523

<https://ovidsp.ovid.com/ovidweb.cgi?T=JS&NEWS=N&PAGE=main&SHAREDSEARCHID=7MqtuNqaQXjovL97Dyjh2Uh2gKf4DvMPrPV2PoPqrAmpx5VeeZZJOqgN0LYX39ulj>

# Results: Titles & Abstracts

Please see attached compressed EndNote library and RIS file.
