# Supplementary material for: Patient and Public Involvement in Inflammatory Bowel Disease Research—A Scoping Review
Source: J Can Assoc Gastroenterol. 2023 Dec 14;7(2):137–48. doi: 10.1093/jcag/gwad054 (PMC10999768; doi:10.1093/jcag/gwad054)
Supplement: gwad054_suppl_Supplementary_Materials [file gwad054_suppl_supplementary_materials.zip › gwad054_suppl_Supplementary_Content_3.docx]

| **Article**  **Supplementary Data Content 3.** Reporting PPIn according to Patient-Oriented Research Level of Engagement Tool (PORLET) criteria (n=49) | **Criterion 1: Patients as Partners** | **Criterion 2: Patient Identified Priorities** | **Criterion 3: Outcomes Important to Patients** | **Criterion 4: Aims to Integrate Knowledge into Practice** | **Criterion 5: Team is Multi-Disciplinary** | **Total Score** |
| --- | --- | --- | --- | --- | --- | --- |
| Adegbola et al^15^ | 4 | 3 | 1 | 4 | 2 | 14 |
| Adegbola et al^16^ | 3 | 3 | 1 | 3 | 2 | 12 |
| Adler et al^17^ | 2 | 4 | 1 | 1 | 3 | 11 |
| Alnaqbi et al^18^ | 1 | 2 | 1 | 1 | 2 | 7 |
| Alrubaiy et al^19^ | 1 | 3 | 1 | 1 | 2 | 8 |
| Bitton et al^20^ | 1 | 3 | 1 | 1 | 2 | 8 |
| Bitton et al^21^ | 3 | 3 | 1 | 1 | 2 | 10 |
| Bodger et al^22^ | 3 | 4 | 1 | 1 | 2 | 11 |
| Carter et al^23^ | 4 | 4 | 1 | 1 | 4 | 14 |
| Casellas et al^24^ | 1 | 2 | 1 | 1 | 2 | 7 |
| Cheifetz et al^25^ | 1 | 3 | 3 | 1 | 2 | 10 |
| de Jong et al^26^ | 4 | 4 | 3 | 1 | 5 | 17 |
| Denters et al^27^ | 1 | 4 | 1 | 1 | 2 | 9 |
| Dibley et al^28^ | 3 | 3 | 3 | 3 | 2 | 14 |
| Dibley et al^29^ | 3 | 4 | 3 | 2 | 2 | 14 |
| Fofaria et al^30^ | 5 | 5 | 4 | 1 | 5 | 20 |
| Grant et al^31^ | 5 | 5 | 5 | 3 | 5 | 23 |
| Guida et al^32^ | 1 | 2 | 1 | 1 | 2 | 7 |
| Haaland et al^33^ | 1 | 2 | 1 | 1 | 2 | 7 |
| Hart et al^34^ | 4 | 4 | 3 | 3 | 3 | 17 |
| Hubbard et al^35^ | 3 | 5 | 1 | 4 | 3 | 16 |
| Hughes et al^36^ | 1 | 3 | 1 | 3 | 5 | 13 |
| Kapasi et al^37^ | 3 | 4 | 3 | 1 | 5 | 16 |
| Katarina et al^38^ | 1 | 3 | 1 | 1 | 3 | 10 |
| Kennedy et al^39^ | 3 | 5 | 1 | 1 | 3 | 13 |
| Kennedy et al^40^ | 3 | 4 | 1 | 1 | 2 | 11 |
| Kennedy et al^41^ | 5 | 5 | 5 | 5 | 2 | 22 |
| Khalil et al^42^ | 3 | 4 | 1 | 3 | 2 | 13 |
| Kim et al^43^ | 1 | 3 | 1 | 3 | 2 | 10 |
| Kim et al^44^ | 4 | 4 | 4 | 3 | 5 | 20 |
| Lee et al^45^ | 1 | 3 | 1 | 1 | 2 | 8 |
| Louis et al^46^ | 3 | 4 | 4 | 3 | 3 | 17 |
| Wickman et al^47^ | 1 | 3 | 1 | 1 | 2 | 8 |
| Macdonald et al^48^ | 4 | 4 | 3 | 4 | 2 | 17 |
| Marín-Jiménez et al^49^ | 4 | 4 | 3 | 1 | 5 | 17 |
| McDermott et al^50^ | 3 | 3 | 3 | 1 | 2 | 12 |
| Oliver et al^51^ | 1 | 3 | 1 | 1 | 3 | 9 |
| Rohatinsky et al^52^ | 5 | 5 | 5 | 5 | 5 | 25 |
| Ruan et al^53^ | 1 | 3 | 1 | 1 | 2 | 8 |
| Vent-Schmidt et al^54^ | 1 | 3 | 1 | 1 | 2 | 8 |
| Vergara et al^55^ | 1 | 2 | 1 | 1 | 2 | 7 |
| Williams et al^56^ | 3 | 4 | 4 | 4 | 3 | 18 |
| Sahnan et al^57^ | 3 | 3 | 3 | 1 | 3 | 13 |
| Heisler et al^58^ | 4 | 4 | 4 | 1 | 3 | 16 |
| Santos et al^59^ | 4 | 4 | 4 | 4 | 4 | 20 |
| Cavallaro et al^60^ | 1 | 1 | 2 | 1 | 2 | 7 |
| Rines et al^61^ | 5 | 5 | 5 | 5 | 5 | 25 |
| Long et al^62^ | 1 | 3 | 1 | 1 | 2 | 8 |
| Gorbenko et al^63^ | 1 | 3 | 1 | 1 | 2 | 8 |
